# Successful true cavity pathfinding with balloon assisted CTO with bifurcation lesions: Two case reports

**DOI:** 10.1097/MD.0000000000037404

**Published:** 2024-03-29

**Authors:** Shichang Zhang, Guangxin Hu, Botao Zhang, Yinping Li, Ben Li, Zhijun Liu, Ping Ma, Yumin Qiu, Qingbin Xu

**Affiliations:** a Department of Cardiology, Cardiovascular and Cerebrovascular Disease Hospital of General Hospital of Ningxia Medical University, Yinchuan, Ningxia, China.

**Keywords:** balloon extrusion, bifurcation lesions, chronic total occlusion, PCI

## Abstract

**Background:**

Coronary artery disease is a prevalent global cardiovascular ailment, with percutaneous coronary intervention (PCI) standing out as a crucial method for relieving symptoms and enhancing the quality of life in patients with coronary heart disease. However, the presence of concurrent chronic total occlusion (CTO) and bifurcation lesions within coronary arteries elevates the complexity and treatment risks, especially when the entry point of the CTO is ambiguous.

**Objective:**

This study aims to present an innovative approach for treating CTO complicated with bifurcation lesions, focusing on true cavity pathfinding assisted by a balloon.

**Methods:**

Two cases of CTO patients with concomitant bifurcation lesions are described. One case involves CTO of the left anterior descending artery) combined with anterior non-angle trigeminal lesions, while the other entails CTO of the posterior left artery combined with posterior angle trigeminal lesions. True lumen identification using a balloon and subsequent opening of the CTO blood vessel were performed in both cases.

**Results:**

In both cases, the true lumen was successfully located with the assistance of a balloon, leading to the successful opening of the CTO blood vessel. This approach not only simplified the procedure but also reduced procedural difficulty and associated risks of complications compared to traditional guide wire operations.

**Conclusion:**

The application of true cavity pathfinding assisted by a balloon offers a novel and effective strategy for managing CTO complicated with bifurcation lesions. The method simplifies the procedure, decreases procedural difficulty, and lowers the risk of complications associated with guide wire operations. However, further studies and long-term follow-up data are warranted to validate the reliability and long-term efficacy of this innovative approach.

## 1. Introduction

Coronary artery disease has always been one of the major cardiovascular diseases in the world, and interventional therapy has become an important means to alleviate symptoms and improve quality of life of patients with coronary artery disease.^[1]^ Compared with coronary artery bypass grafting (CABG), percutaneous coronary intervention (PCI) has several advantages, including faster recovery, more direct clinical improvement, higher success rate, and lower postoperative mortality.^[2]^ However, in cases where chronic total closure of the coronary artery (CTO) co-exists with bifurcation lesions, the difficulty and risk of treatment is significantly increased, especially when the location of the CTO entrance is unclear. In recent years, the continuous development of coronary intervention technology has provided new possibilities for the management of such complex cases.

The PCI strategy of CTO has become a mature treatment strategy for patients with specific chronic coronary syndromes.^[3]^ Thanks to technological advances, standardized algorithms, and improved operator expertise, the success rate of CTO procedures has increased significantly over the past decade from 50% to 70% to 85% to 94%.^[4]^ However, CTO with bifurcated lesions is a major challenge in coronary interventional therapy. The difficulty of treatment lies not only in the particularity of CTO itself, but also in the complexity introduced by bifurcated lesions. Bifurcated coronary artery disease is common because changes in endothelial shear stress promote the development of atherosclerosis.^[5]^ Coronary artery bifurcation shows local turbulence and enhanced atherosclerotic thrombosis, platelet deposition, and plaque rupture.^[6]^ A variety of anatomical and physiological factors should be considered in the treatment of bifurcation lesions. The presence of bifurcation lesions will not only increase the difficulty of operation, but also may lead to changes in hemodynamics and increase the risk of postoperative complications.^[7]^ In particular, when the location of the CTO entrance is not clear, the traditional guidance line operation may further increase the uncertainty of treatment. Previous studies have found that about 20% of patients with coronary angiography have CTO,^[8]^ and the incidence of CTO combined with bifurcation lesions is as high as 25%.^[9]^ PCI technology has been continuously developed and improved in recent years, and the success rate of the procedure has been greatly improved, and it has been reported that the success rate of experienced centers has already reached 90%.^[10]^ However, the combination of CTO with bifurcation lesions has undoubtedly increased the complexity of the intervention and the perioperative risk, and the amount of intraoperative radiation, contrast use, and procedure time are significantly increased compared with that of ordinary CTO.^[9]^ perioperative risks, and the amount of intraoperative radiation, the amount of contrast used, and the duration of the procedure are all significantly increased compared with normal CTO.^[9]^ The presence of bifurcation lesions also reduces the success rate of the procedure and increases the risk of pericardial tamponade after guidewire perforation and post-interventional side branch loss.^[11]^ Therefore, by analyzing and introducing 2 cases of CTO complicated with bifurcated lesions, this paper aims to explore a relatively fast, effective and safe new technology for opening CTO, with a view to providing a feasible plan for the treatment of such patients.

## 2. Case information

This study was approved by the Ethics Committee of Cardiovascular and Cerebrovascular Disease Hospital of Ningxia University General Hospital. Written informed consents were obtained from the guardians in this study.

### 2.1. Case 1: Chronic total occlusion combined with anterior non-angular trigeminal lesions

The patient was a 73-year-old man who presented to the Cardiovascular and Cerebrovascular Disease Hospital of Ningxia University General Hospital with the complaint of “intermittent chest pain in the past week, which lasted for 4 days and gradually worsened.” The patient had a history of hypertension for 30 years, but denied having diabetes. It is worth noting that the patient underwent “CABG” in January 2015 at an outside hospital, and the specific details of the surgery are not known. In addition, the patient’s personal history included a history of smoking for more than 40 years, with an average of approximately 20 cigarettes per day.

Specialized physical examination showed that the patient’s blood pressure on admission was 149/86 mm Hg, heart rate was 68 beats/min, heart rhythm was Qiqi, no murmurs were heard in the valvular regions of the heart, and the respiratory sounds of both lungs were clear, and no dry or wet rales were heard. On admission, the results of ancillary tests showed an NT-ProBNP level of 759.0 pg/mL, an LDL cholesterol level of 2.31 mmol/L, and a normal troponin I test result. The electrocardiogram showed sinus rhythm (70 beats/min) and mild ST-segment depression (0.1–0.2 mV) in leads I, aVL, and V1–V4. Cardiac color Doppler ultrasound showed a left ventricular ejection fraction of 64.73% and a ventricular wall motion fraction of 35.42%. In addition, the patient had undergone CABG, which resulted in an enlarged left atrium of 39 mm and reduced left ventricular diastolic function but normal left ventricular systolic function. Based on the results of the admission examination, the patient was diagnosed with the following diseases: coronary artery disease with unstable angina pectoris, which had been treated with CABG; hypertension (grade 2, which is a high-risk category); coronary artery myocardial bridge.

**Figure 1.**
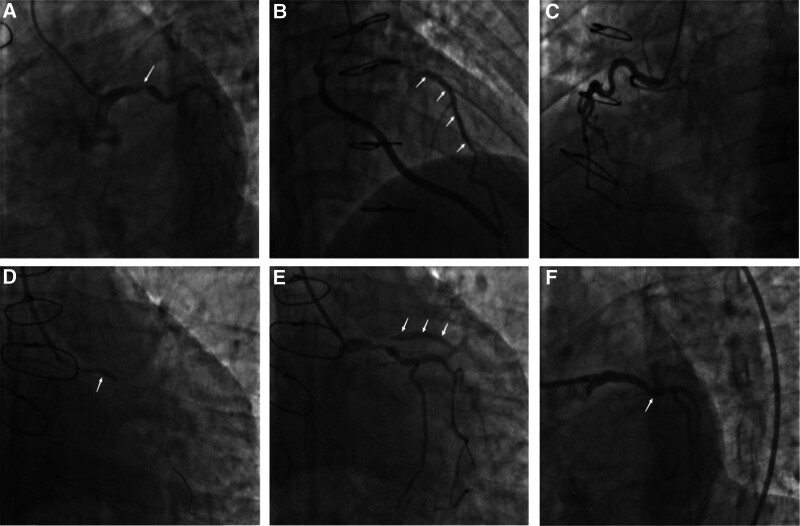
Angiographic image of case 1. (A) left coronography (spider): LM end and LCX opening were severely narrowed, and no stump occlusion was found in the LAD (B) bridge angiography (right shoulder): bilateral anastomosis and bridge vessels were patency without stenosis (C) right coronary angiography (left anterior oblique): No significant stenosis of the RCA lumen (D) balloon extrusion: The OMNIPASS balloon (2.0 mm × 20 mm) expanded the LM end to the LCX opening once (foot position) at 16 atm (E) postprimary balloon compression (foot): Proximal imaging of the previously occluded LAD (F) post-PCI imaging (spider position): LCX-LM was implanted into the PROMUS PREMIER stent (3.5 mm × 12 mm). LCX = left circumflex branch , LCX-LM = left circumflex branch-left main, LM = left main, LAD = left anterior descending artery.

After admission, the patient was actively treated with conventional secondary prevention medications for coronary artery disease, and Coronary arteriography and PCI were planned after contraindications to surgery were ruled out. For the patient’s detailed operative record, please refer to Figure 1 A~F.

### 2.2. Case 2: Chronic total occlusion combined with posterior trigeminal angular lesion

The patient, a 56-year-old male, was referred to the Cardio-Cerebrovascular Disease Hospital of Ning Medical University General Hospital because he complained of “recurrent episodes of chest pain in the past 3 years, the most recent episode was 1 week ago.” The patient had a history of high blood pressure for up to 3 years and also had diabetes for 3 years. In terms of personal history, the patient had a long history of smoking, with a smoking history of 30 years and an average of 30 cigarettes per day.

In the specialized physical examination, the patient’s blood pressure at admission was 120/70 mm Hg, the heart rate was 78 beats/min, no murmurs in the heart valve area were found during auscultation, and the respiratory sound was clear during the double lung auscultation, and no dry and wet rales were heard. The results of the auxiliary examination on admission showed a low density lipoprotein cholesterol level of 2.31 mmol/L, while the test results of NT-ProBNP and troponin I were not abnormal. The ECG showed sinus rhythm (80 beats/min), and the ST-segment of the II, III, aVF, and V2–V4 leads was slightly depressed (0.05–0.1 mV). The left ventricular ejection fraction and wall motion fraction were 58.77% and 31.67% respectively. In addition, the left ventricular end-diastolic diameter was 60 mm. The patient had undergone PCI surgery, had mild aortic regurgitation, decreased left ventricular diastolic function, but normal left ventricular systolic function. According to the results of admission examination, the patient was diagnosed with the following diseases: coronary heart disease, unstable angina pectoris, and had undergone PCI; enlargement of the heart, mainly involving the left ventricle; hypertension (level 3, high-risk category); type 2 diabetes.

**Figure 2.**
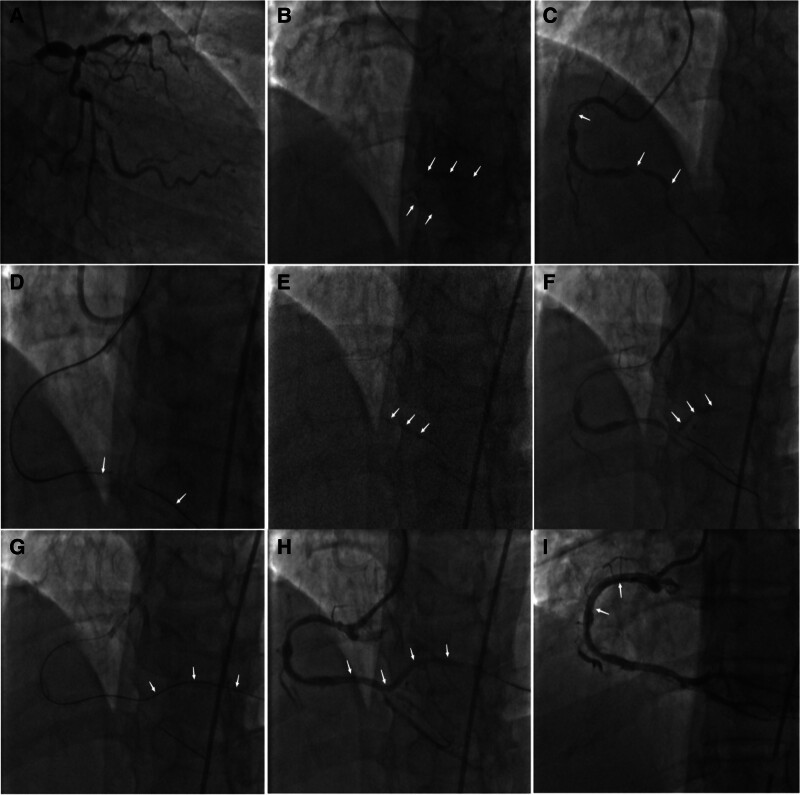
Angiographic image of case 2. (A) left coronary angiography (liver): LM, LAD, LCX showed serious lesions (B) left crown reverse perfusion right crown angiography (left anterior oblique): PLA distal segment and individual branches were developed (C) right coronography: Severe lesions can be seen in the proximal and distal segments of RCA, without stump occlusion in PLA (D) forward attempts of multiple CTO guides into the block section with the assistance of double-cavity microcatheter failed (E) balloon extrusion: Pioneer balloon (1.5 mm × 15 mm) was used to dilate the lesions at the mouth and bifurcation of small vessels near PDA twice at 16 atm pressure (F) angiography after initial balloon extrusion: PLA was developed and TIMI3 blood flow was restored (G) the positive guide wire successfully reached the distal end of PLA with the assistance of a two-cavity microcatheter (H) FIREBIRD2 stent placement (3.0 mm × 29 mm) in PLA proximal segment to RCA distal segment (I) a Helios stent (4.0 mm × 38 mm) was inserted into the proximal segment of the RCA. CTO = chronic total occlusion, LAD = left anterior descending artery, LCX = left circumflex branch, LM = left main, PDA = posterior descending artery, PLA = posterior left artery, RCA = Right Coronary Artery, TIMI = thrombolysis in myocardial infarction.

Upon admission, the patient was actively treated with conventional secondary preventive drugs for coronary heart disease, and Coronary arteriography and PCI were planned after contraindications for surgery were ruled out. Detailed surgical records are shown in Figure 2A~I.

## 3. Results

In the first case, the patient was successfully treated with true cavity path planning assisted airbags to locate the true cavity and successfully open the CTO vessel. This approach not only simplifies the procedure, but also reduces the difficulty and risk of complications associated with traditional wire guide operations. The patient did not have any symptoms of chest pain after surgery, and the follow-up showed that the symptoms were effectively controlled.

In the second case, the patient was also successfully treated with a CTO using true cavity path planning assisted balloon to locate the true cavity and open the vessel. After surgery, the patient reported significant relief from chest pain and plans to further manage issues related to the left crown 1 month later.

## 4. Discussion

Interventional treatment of bifurcation lesions, especially complex bifurcation lesions, has always been an important challenge faced by cardiac interventional specialists. With the continuous progress of technology and materials, we are expected to improve the prognosis of patients after interventional treatment of bifurcation lesions.^[12]^ Some studies have indicated that technical complexity, failure of retrograde approach or imperfect revascularization in PCI therapy of CTO are the main challenges at present. In order to reduce the incidence of subintimal hematoma, some technologies and devices can release hematoma after the formation of hematoma, and balloon occlusion is one of the strategies.^[13]^ The use of drug-coated balloon to treat bifurcated lesions is a new therapeutic method.^[14,15]^

This article deals with 2 patients who both suffered from CTO accompanied by bifurcation lesions. One patient’s CTO was located in the left anterior descending artery (LAD) with anterior non-angle trifurcation lesions, while the other patient’s CTO was in the posterior left artery (PLA) with posterior angle trifurcation lesions. By lateral branch contrast examination, it was confirmed that the occluded segments of the CTO in these 2 patients were relatively short, with the possibility of positive opening. However, the difficulty lies in the fact that the fiber caps proximal to the vessels in both patients were not clear enough, which greatly increased the difficulty of guiding the guidewire into the true lumen. According to the current CTO treatment technology, we have appropriate management strategies for this type of lesion. The preferred approach for CTO lesions without stumps, with ill-defined positive entrances, or with occlusion after branch vessel emanation is to use intravascular ultrasound -guided true lumen tracing technique.^[16–21]^ Of course, in addition to finding the true cavity with the support of imaging, the occlusion of blood vessels can also be opened by “ADR, BASE, Carlino, and other” forward techniques. If the conditions of the reverse collateral are good, the operation can also be completed by reverse technology.^[22–24]^ However, it is important to note that subintimal tracking, especially with ADR (lead-based or device-based) methods, often increases the risk of vascular dissection, and intraplaque tracking can also lead to vascular dissection because the specialized CTO guides are very rigid and designed to traverse CTO plaques, making it difficult to precisely control the lead position. This may lead to complications.^[25,26]^

Therefore, for these 2 patients mentioned in our paper, we used a special approach to achieve the goal of true lumen pathfinding. In case 1, the patient had been treated with a LAD coronary artery bypass surgery 7 years earlier at an outside hospital. However, during the current coronary angiography, we found that the previously constructed bridge vessel was from the patient’s artery and that there was no stenosis in the bilateral anastomoses as well as in the bridge vessel. Based on the angiographic findings, we decided to treat the lesions in the left main and left circumflex branch. After the guidewire successfully traversed the left circumflex branch lesion and reached the distal end, we prepped the target lesion using a preexpanded balloon according to routine procedures. When the contrast was performed again, the visualization of the proximal segment of the LAD became very clear. In terms of surgical strategy, we had 2 options: either to implant a double stent in the anterior trifurcation of the left main trunk or to intervene only in the left circumflex branch-left main lesion. Considering the good condition of the patient’s arterial bridge vessels, we finally chose to implant a stent at the end of the left circumflex branch-left main lesion, and the outcome of the procedure was very satisfactory.

In case 2, the patient suffered from a lesion at the posterior trifurcation with a CTO of the PLA. Left coronary retroperfusion visualization showed a relatively short occluded segment. Initially, we tried various CTO guidewires assisted by single-lumen and double-lumen microcatheters, but none of them failed to breach the mouth of the occluded segment. Inspired by the experience of case 1, we tried to guide the guidewire into the branch closest to the mouth of the PLA occluded vessel, and then used a balloon (1.5 mm × 15 mm) that matched the diameter of the vessel in that branch. We released the dilated balloon twice at a pressure of 16 atm in the proximal segment of the branch vessel to the posterior trigeminal vascular lesion. When imaging was performed again, we found that the proximal segment of the PLA was clearly visualized and almost formed a 90° angled lesion. Finally, with the assistance of a dual-lumen microcatheter, we successfully guided the guidewire to the distal PLA and completed stent implantation without difficulty. Of course, dual-lumen microcatheters often play a crucial role in combined bifurcation of the CTO,^[27]^ Microcatheters are gradually being widely used as coronary intervention tools for the treatment of complex coronary interventions. Selection of appropriate crossover technique using different microcatheters can improve the success rate of PCI, reduce procedure time and contrast use, and decrease radiation.^[28]^ A report stated^[13]^ that a balloon and microcatheter or dual-lumen microcatheter were placed at the proximal coronary CTO lesion, then the balloon was dilated next to the catheter, and most of the collateral blood flow was sealed, thus reducing the possibility of hematoma formation.

In this paper, by analyzing the above 2 cases of balloon assisted CTO combined with bifurcation lesions in which true lumen pathfinding was successfully performed, we hypothesize that balloon dilatation of the bifurcation may have led to vascular visualization of the occluded segment because of the fragmentation and displacement of plaque by the squeezing action of the balloon, which led to the formation of microchannels. Of course, the relatively short occluded segments in these 2 CTO patients may also have contributed to the visualization effect after balloon extrusion. Therefore, it is reasonable to believe that this may be a new strategy for true lumen pathfinding in CTO combined with bifurcation lesions, which is potentially applicable to both angulated and non-angulated lesions.

**Figure 3.**
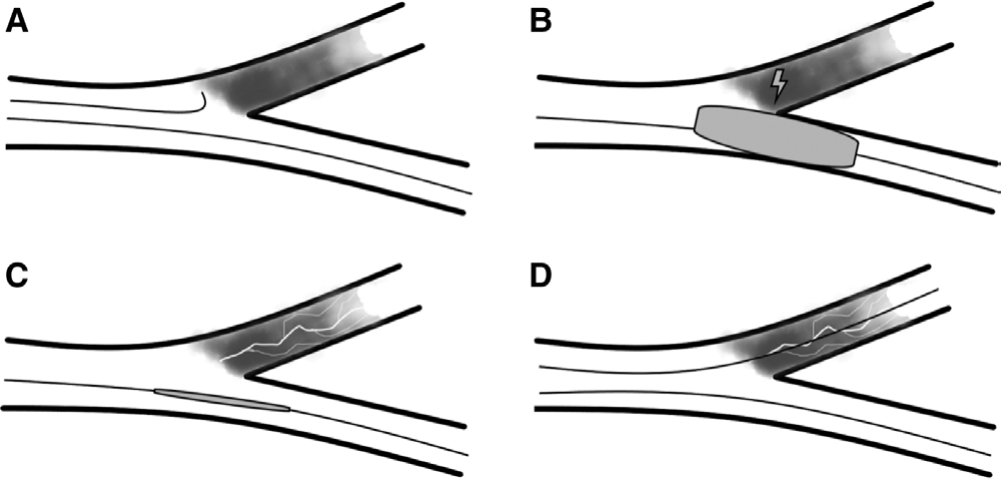
Operation steps of the “balloon extrusion and fragmentation” technique.

The main steps of this “balloon extrusion and fragmentation” technique are as follows: When the bifurcation has no stump and the positive guidewire cannot pass through it (Fig. 3A). The selection of an extrusion balloon tailored to fit the side branch vessels and its placement within the bifurcation for expansion (Fig. 3B). Following balloon extrusion, the plaque undergoes fragmentation, resulting in the formation of a microchannel. Blood flow is then restored to the CTO vessel from the proximal segment, confirmed through angiography to verify the reestablishment of blood flow (Fig. 3C). An orthogonal guidewire can reach the true lumen of the distal CTO vessel via the microchannel (Fig. 3D).

In summary, the experience of successful true lumen pathfinding with this technique includes the following:

CTO anatomical basis: this technique is applicable to the specific anatomical basis of the CTO, including the absence of stumpy occlusion at the bifurcation, the presence of extruded vessels adjacent to the mouth of the occluded vessel that can accommodate the smallest balloon, and relatively short occluded segments as confirmed by ipsilateral or contralateral retrograde perfusion visualization.

Selection of extrusion balloon: the selection of extrusion balloon needs to be determined according to the lesion of the mouth. If there is a lesion at the mouth of the extruded vessel, it is recommended that the ratio of the balloon diameter to the vessel diameter be 1:1; if there is no lesion at the mouth, it is recommended that the ratio be 1.25:1. The pressure released by the balloon should be sufficient to form an effective extrusion, but it should not trigger vascular entrapment.

Guidewire selection: if microchannels are formed after squeezing the balloon, the preferred guidewire should have a hydrophilic coating and try to pass through the microchannels, which can be upgraded or assisted by a microcatheter if necessary.

The shortcoming of this article is that 2 of the patients in this article did not undergo intravascular ultrasound and/or optical coherence tomography in time for the restoration of blood flow in the occluded vessel to clarify the morphology and nature of the plaque. We hope that the “balloon compression plaque fragmentation” technique will be widely disseminated for use by interventionalists, and that its effectiveness and safety will be verified through more clinical case summaries and studies.

## 5. Limitations

Limitations of this study include the limited sample size and the fact that it was based on only 2 specific cases, left anterior descending artery total occlusion and posterior left artery total occlusion, making it difficult to generalize to a wider patient population. The lack of a control group challenges the accurate assessment of the relative effectiveness of the new approach versus conventional wire guide manipulation, and the study did not provide detailed data on long-term effects and treatment outcomes, and more long-term follow-up data is needed to verify the persistence and long-term efficacy of the new approach. In addition, the study only focused on a specific type of complication, that is, CTO accompanied by double-fork lesions, while the applicability of other complications or case types has not been studied in depth. Therefore, in order to fully evaluate and confirm the effectiveness and feasibility of this innovative approach, further studies are needed to cover more patient populations and contexts.

## 6. Conclusion

The application of a therapeutic method that realizes true lumen pathfinding with the aid of balloon assistance provides a new idea for the treatment of CTO combined with bifurcation lesions. By avoiding the possible risks associated with guidewire manipulation, we have not only simplified the surgical steps, but also reduced the difficulty of the operation and the risk of complications. Nevertheless, we also recognize that further studies and long-term follow-up data are still necessary to further validate the reliability and long-term efficacy of this method.

## Acknowledgments

Thanks to the support of the universal-level project of Ningxia Medical University (No. XM2022028).

## Author contributions

**Conceptualization:** Shichang Zhang, Guangxin Hu, Botao Zhang, Yinping Li, Ben Li, Zhijun Liu, Ping Ma, Yumin Qiu, Qingbin Xu.
